# Harnessing the pillars of institutions to drive environmentally sustainable hospital foodservices

**DOI:** 10.3389/fnut.2022.905932

**Published:** 2022-09-12

**Authors:** Stefanie Carino, Jorja Collins, Shirin Malekpour, Judi Porter

**Affiliations:** ^1^Department of Nutrition, Dietetics and Food, Monash University, Notting Hill, VIC, Australia; ^2^Dietetics Department, Eastern Health, Box Hill, VIC, Australia; ^3^Monash Sustainable Development Institute, Monash University, Clayton, VIC, Australia; ^4^School of Exercise and Nutrition Sciences, Institute for Physical Activity and Nutrition (IPAN), Deakin University, Geelong, VIC, Australia

**Keywords:** foodservice, environmental sustainability, hospitals, change, qualitative study

## Abstract

**Background:**

The cultural-cognitive, normative and regulative pillars of institutions influence the ability of hospitals to change how they function at an organizational and operational level. As more hospitals and their foodservices instigate changes to address their environmental footprint and impact on food systems, they move through the “sustainability phase model” from no response through to high level action and leadership. The aim of this study was to describe and compare the pillars of institutions between hospitals in different stages of achieving environmentally sustainable foodservices (business-as-usual vs. exemplar hospitals).

**Methods:**

For this qualitative inquiry study, interviews were conducted with 33 hospital staff from 3 business-as-usual hospitals in Melbourne, Australia and 21 hospital staff from 14 exemplar hospitals across 9 countries. Participants were asked questions about their perspectives on environmental sustainability in foodservices and the barriers, enablers and drivers they experienced. Each data set was analyzed thematically and then compared.

**Findings:**

There was a clear and distinct difference in responses and behaviors within each pillar between the exemplar and business-as-usual hospitals. The cultural-cognitive pillar identified a similarity in personal belief in the importance of addressing environmental impacts of foodservices, but difference in how staff saw and acted on their responsibility to drive change. The normative pillar uncovered a supportive culture that encouraged change in exemplar hospitals whilst business-as-usual hospital staff felt disheartened by the difficult processes and lack of support. The regulative pillar reflected business-as-usual hospital staff feeling restricted by government policy vs. exemplar hospital participants who were motivated to internalize government policy in different ways and work with other hospitals to advocate for better policy.

**Interpretation:**

These findings highlight strategies related to each of the three pillars of institutions that can be used to drive effective, sustainable long term change within hospitals. This includes staff education and training, revisiting hospital culture and values around environmental sustainability, embedding sustainable foodservices in internal policies, and a comprehensive government policy approach to sustainable healthcare.

## Introduction

Despite its vital role in protecting human health and well-being, the healthcare industry can have vast negative impacts on the environment (1). This in turn has negative effects on human health, setting up a paradoxical cycle. Hospitals in particular use large quantities of natural resources and produce high volumes of waste (2). Internationally, healthcare institutions including the National Health Service in the United Kingdom are recognizing their responsibility to address their environmental footprint and are committing to carbon neutral healthcare (3).

Alongside this reform, food system transformation is urgently needed. The recently released Intergovernmental Panel on Climate Change has described the current and worsening impacts of climate change on food production and food security (4). Food provided to hospitalized patients contributes to this environmental footprint. Hospitals typically provide three meals, snacks and drinks to all patients every day. Inputs such as water, fuel, gas, electricity and land are required to grow food and manage the production system to produce this food, whilst waste is one of the major negative outputs produced throughout the supply chain. However, creating the type of change that is necessary for hospitals can be difficult as multiple barriers exist to systems change (5).

The “sustainability phase model” is a tool designed to assess and compare organizations' commitment to environmental sustainability (6). It describes six phases organizations may move through on their improvement trajectory. In the early stages of the model, the organization begins with a disregard for their negative environmental impacts, a lack of awareness of action to be taken and then starts to implement environmentally sustainable practices but with a focus on image building, reducing cost and increasing efficiency. In the later stages of the model, sustainability becomes part of the business strategy and the organization becomes “strategically proactive” or a “sustaining organization” where they co-operate with other organizations for broader societal transformation. This process may not necessarily occur linearly, as some organizations may advance quickly through some phases, or regress back to previous practices. This model has been previously used to study how sustainable change evolves within various industries (7).

The way hospitals achieve environmentally sustainable foodservices varies and is reliant on several internal and external factors (8). The functioning of institutions (including hospitals) has been described as being dependent on three pillars: regulative, cultural-cognitive and normative (9). The “cultural-cognitive” pillar describes the beliefs and knowledge of the people within institutions, the “normative” pillar considers the ethics, values and personality of the institution itself, and lastly the “regulative” pillar comprises the laws, regulations and policies that govern the institution. These pillars of institutions would look different in hospitals with environmentally sustainable foodservices at the stage of a “sustaining organization” compared to hospitals in earlier stages of the sustainability phase model. These three pillars working concurrently may facilitate the multi-level change needed within organizations to move them through the sustainability phase model. The pillars of institutions framework has been used as an analytical tool for sustainable activities in institutions. For example, a study investigating the institutional drivers for a circular economy amongst institutional environments in China, Europe and the US (10). As such, the aim of this study was to describe and compare the pillars of institutions between hospitals in different stages of achieving environmentally sustainable foodservices (business-as-usual vs. exemplar hospitals).

## Methods

This study was approved by the Monash University Human Research Ethics Committee (Project ID: 24912 and 19730) and Eastern Health Human Research Ethics Committee (Reference number: LR19/025).

### Research design

This study used a generic qualitative inquiry approach, seeking to understand a real world problem through participants' response to a series of open ended and practical questions (11–13). The “sustainability phase model” was used to guide the classification of hospitals at different stages of change. The inquiry sought to analyze two sets of pre-existing interview data from these hospitals, to describe and compare the cultural-cognitive, normative and regulative pillars of institutions.

### Participants

This study used two pre-existing data sets. As both data sets had been previously analyzed and published, there was clear evidence of their stage of the sustainability phase model. The original settings and participants were purposefully selected. The first data set was obtained through interviews with staff from “business-as-usual” hospitals. Originally, this research sought to capture broadly the staff perspectives toward environmentally sustainability in foodservices. The hospitals were part of a large metropolitan public healthcare network in Melbourne, Australia. These hospital sites were convenience sampled due to existing relationships and selected as they had previously been part of a larger food waste audit. Staff who participated in interviews worked across the food supply chain contributing to patient food provision and were in leadership positions, sustainability roles, procurement, foodservice, nursing or dietetics (14). This research has been previously published, with the results of the staff interviews revealing that these hospitals were practicing at the lower end of the sustainability phase model (14). This was characterized by a view of environmental sustainability as an exercise for compliance, reputation and cost savings (6, 14).

The second data set was obtained through interviews with staff from purposefully selected “exemplar” hospitals. The exemplar hospitals were identified as such for having well-established environmentally sustainable foodservice practices and worked to influence other hospitals. Their “exemplar” status was evidenced by them having received relevant awards, featured in webinars, reports, and publications for their success. The hospitals were a combination of public and private hospitals. The hospital contact initially contacted was asked to identify the staff member(s) with the most extensive knowledge about the sustainable foodservice practices, such as foodservice staff, dietitians or hospital executive (8).

### Data collection

Data collection procedures for both data sets have been previously published (8, 14). In brief, for the business-as-usual hospitals, semi structured individual or small group interviews were conducted in person in July to November 2019. Eligible participants were known to the research team or identified through professional networks, as well as snowball sampling used to identify additional participants. Small group interviews were used for participants where there are multiple people in that role, for example nurses, dietitians, foodservice staff. Participants were asked to share their perspectives on environmental sustainability in foodservices and to provide recommendations to aid change. The number of participants interviewed was deemed sufficient using the concept of “information power.” Information power guides sample size for qualitative studies and indicates that the more information a sample holds, the less participants are needed, which depends on the study aim, sample specificity, use of established theory, quality of dialogue and analysis strategy (15). Therefore information power was considered throughout recruitment and considered complete once perspectives were captured from a variety of staff roles and the original research question could be answered.

For the exemplar hospitals, semi structured individual or small group interviews were conducted *via* Zoom in October 2020 to January 2021. Participants were asked to share their hospital's environmentally sustainable practices and the drivers and enablers of these. The number of hospitals recruited was also guided by “information power” whereby this concept was considered throughout recruitment and was complete when study aims had been fulfilled and the sample included hospitals from a variety of contexts. For both data sets, the aim of recruitment was not necessarily a thoroughly representative sample, but to capture a diverse range of perspectives to be able to sufficiently answer the original research question. For both data sets, all interviews were conducted, recorded and transcribed by the same researcher.

### Data analysis

Both datasets were recoded using a deductive approach with a predefined coding framework based on the cultural-cognitive, normative and regulative pillars of institutions. During coding, quotes from interviews that aligned with themes were identified, and later considered by the research team to include in the results section. Coding with this framework was conducted using NVivo (Release 1.3). A second researcher verified a sub-section of coding within the framework. Once data was organized within the pillars of institutions framework, it was analyzed thematically using the process described by Braun and Clarke (16). This was completed separately for one data set and then the other, and then comparisons were made between themes in the two data sets. This was achieved by creating a table of points underneath each pillar for the two data sets to illuminate key differences.

## Results

From three business-as-usual hospitals a total of 33 participants were interviewed across 11 individual and 7 small group interviews of 2 to 5 participants. The mean interview duration was 22 min. These hospitals were located in Melbourne, Australia and were general public hospitals. There were 14 exemplar hospitals across 9 countries, with a total of 21 participants interviewed. The mean interview duration was 70 min. These hospitals were located in Australia, Singapore, Taiwan, Canada, Denmark, the United States of America, Austria, the United Kingdom and Netherlands, and were a combination of public and private general and women and children's hospitals. Further descriptive information of the demographics of the hospitals and participants is detailed in the initial publications of these results (8, 14).

**Figure 1:**
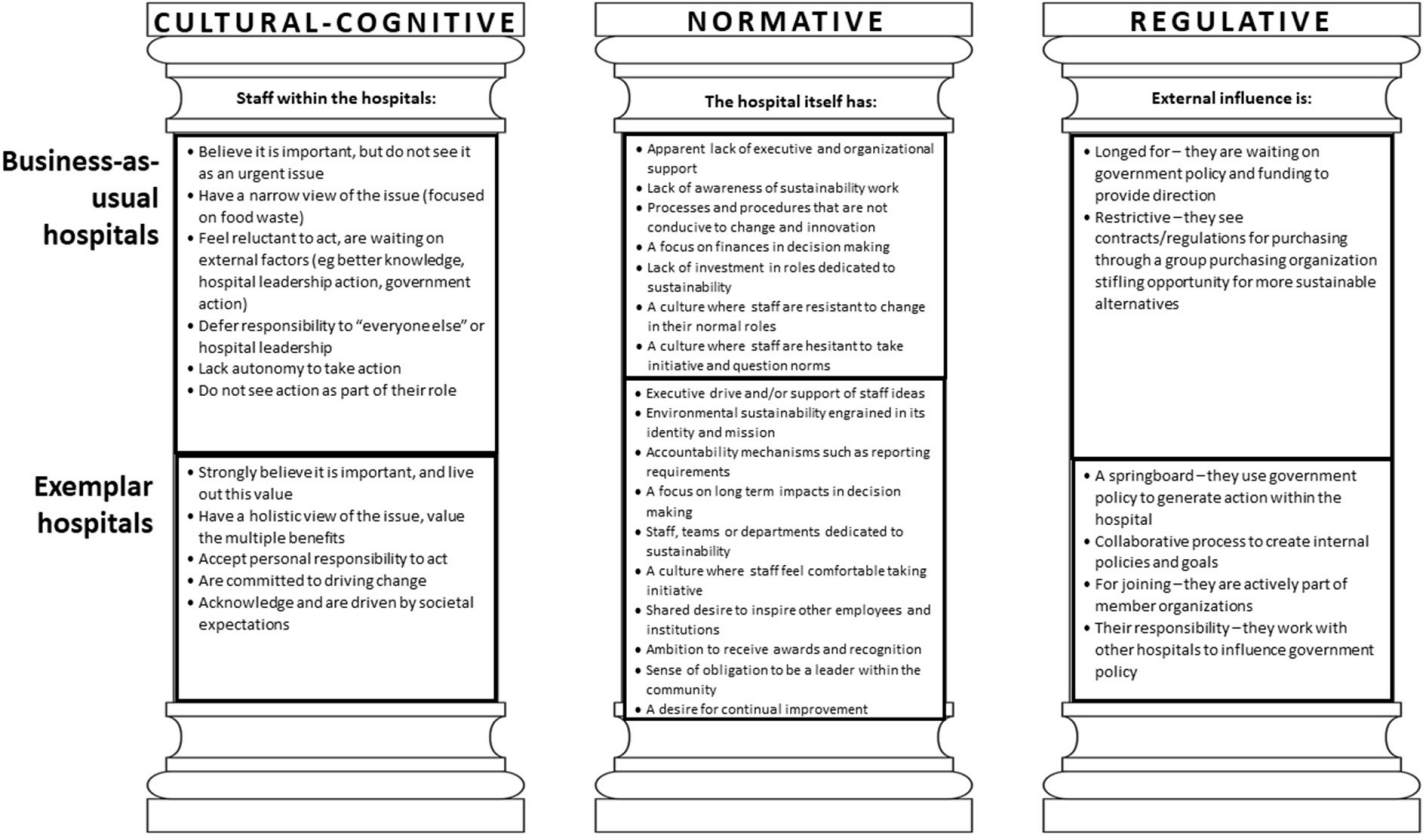
Staff perspectives toward environmental sustainability of hospital foodservices aligned to the pillars of institutions.

There was a clear and distinct difference in responses and behaviors across each pillar. A summary of the perspectives of staff related to each pillar are outlined in Figure 1.

### Cultural-cognitive pillar

Clear similarities and differences in how staff act on their personal beliefs and see their role were evident within the cultural-cognitive pillar. Participants from both the business-as-usual hospitals and exemplar hospitals agreed that it is important for hospitals to reduce the environmental impacts of their foodservices. They shared and were open about their personal concern for the environment, for example:

“*For me personally, it is not pumping methane gas into the atmosphere and heating up the planet for my kids, like that is my number one driver” (P1, exemplar hospital)*

Participants from exemplar hospitals acknowledged competing priorities within the workplace, but were committed to working around these so that they did not create a barrier to the urgent action needed. In contrast, participants from business-as-usual hospitals focused on competing priorities and time constraints and were often overwhelmed and overcome by these barriers.

Differences in perspectives about staff and organizational responsibility and capability to act on their beliefs were evident between the two datasets. Participants from business-as-usual hospitals tended to defer responsibility to “everyone else” or hospital leadership, and indicated that they required and were waiting on greater resources to be able to act.

“*It's got to be driven from the top and it will end up feeding through to everyone, and once that happens, I think we're a lot better than what we were” (P18, business-as-usual hospital)*

In comparison, participants from exemplar hospitals believed that they have a personal responsibility to act, even if not defined or expected in their role. Additionally, there was a clear difference in the scope and magnitude of how staff from the two groups of hospitals viewed environmental sustainability in hospital foodservices. Participants from business-as-usual hospitals tended to focus on food waste during the narrative on sustainability issues. In comparison participants from exemplar hospitals had a more holistic view of sustainability, encompassing issues across the wider food supply chain from food production to waste management. Because of this broad outlook, they could see change had multi-level benefits including environmental, economic, organization reputation and patient care benefits.

### Normative pillar

The normative pillar reflected the differences in the internal systems and culture embedded in the organization. Participants from business-as-usual hospitals described a lack of organizational support and reflected on the difficulty of raising ideas where there were long, arduous processes to implement change, and where financial viability was the main priority in decision making. They felt disheartened and discouraged by this. For example:

“*We don't have direction from above from the board or executive to drive that, we have to beg and ask and then maybe get it and likewise if we want to actually push something through we need executive support to support what we want to do” (P8, business-as-usual hospital)*

Participants tended to believe that there was nothing more they could do in their roles to introduce food sustainability initiatives without significant extra time provided.

Participants from exemplar hospitals shared that hospital executive either drove some initiatives or were supportive of staff ideas. Staff felt comfortable to raise ideas with their seniors, in spite of these challenging their current norms. Whilst financial viability was considered by these hospitals, decision making was guided by long term thinking and their goals and values as an organization and the legacy they wanted to have.

“*We're lucky to have our management support to pursue these sorts of things because I think it does pay long term dividends for the health of our community.” (P9, exemplar hospital)*

There was a greater presence of working groups, committees and staff members in dedicated sustainability roles in the exemplar hospitals. Participants recognized their position as an anchor institution in the community and felt obligated to lead by example. Whilst achieving a lot of success, these hospitals acknowledged that they still have room for improvement and desire for continual growth and learning from other hospitals. They were driven to make changes for the positive influence they could have on other hospitals, for example:

“*We wanted to make a systems change. It wasn't just about adding more local food to a menu, which we knew we could do. It was making that system change that was going to impact other health care facilities or group purchasing organization, you know, draw in our manufacturers and distributors and so forth so that they understood what we as an organization, you know, we're looking at in terms of procurement” (P7, exemplar hospital)*

### Regulative pillar

Participants from business-as-usual hospitals shared the belief that the government must create specific policy and provide associated funding for the hospital to take action.

“*I think it should be a state government health initiative, they should be the ones driving that, they're the ones who are giving the money out” (P21, business-as-usual hospital)*

Staff described feeling restricted by existing requirements, for example the requirement to purchase through a group purchasing organization. There was a lack of autonomy or drive to consider how they could influence policy.

In contrast, participants from exemplar hospitals internalized government policy more ambitiously, for example through initiating audits and quality improvement enterprises, creating their own internal policies and goals to exceed government set targets, and seeking additional funding and grants. Participants at these hospitals acknowledged the limitations of these policies and were motivated to work with other hospitals and policy makers to improve and mandate policy.

“*What we really wanted them to do was, what we said is an act, a local food act so it was there, but it wasn't it wasn't law. And that's what we were the group was kind of the collaborative group was working on and we had a couple of government officials and the greenbelt fund and so forth that had worked with us on you know kind of working with the government. We did letters we shared case studies and all kinds of stuff. And they did end up enacting the local food act which is great” (P7, exemplar hospital)*

## Discussion

The pillars of institutions provides a useful framework that highlights the similarities and differences between hospitals at different stages of achieving environmentally sustainable foodservices. The findings suggest that a multi-level and multi strategy approach addressing cultural-cognitive (staff factors), normative (hospital factors) and regulative (external influences) pillars may be helpful to move healthcare organizations along the sustainability phase model, toward exemplar institutions.

The cultural-cognitive pillar uncovered a difference in perspectives about responsibility between the two groups of hospitals. There is a need to leverage and enhance people's potential to drive change. To optimize the cultural-cognitive pillar of institutions, environmental sustainability in healthcare needs to be taught both in tertiary education as well as provided in professional development opportunities. This includes those already in the workforce, such as those in foodservice, dietetic and leadership positions. The limited opportunities specifically for nutrition professionals to learn about sustainability has been previously documented in the literature (17, 18). Sustainable healthcare education develops students' knowledge, skills and attitudes about the interdependence of climate, ecosystems and health, as well as the healthcare sector's environmental impact and provides practical solutions to support ecosystems and human health (19–21). Primarily, education needs to empower learners to embrace their professional duty of resource stewardship and environmentally preferable practice (22).

As described by participants, it is their personal beliefs and concern for the environment which drives their action. This is an important aspect of education and training. Sustainable healthcare education is not just about providing learners with knowledge about the urgency of the issue, it includes developing the values, mindset and agency needed to be proactive in advocating for and creating change (23). This was evident in the exemplar hospitals. Education and training on communicating the benefits of sustainable practice, tailored to the relevant audience is crucial. For example, finance directors and chief executives can be more interested in the financial and reputational benefits of sustainable changes (24). This effective communication is an important skill to harness and implement. Establishing high quality sustainable healthcare education requires multi-level leadership and collaboration, privileging student voices, developing a sustainable healthcare education curriculum and resources, and integration into course accreditation standards (25).

The normative pillar revealed the value of an organizational vision and healthcare system that recognizes the importance of environmental sustainability at the hospital level, and within the roles of healthcare staff. There is a need to revisit the traditional values of healthcare that center on safety and quality of clinical care, and realign them so that planetary health is at the forefront of health service planning (23). Planetary health is defined as both the health of human civilization and the state of the natural systems on which it depends (26). Historically, climate change has been framed as an external, technical challenge (27). A new discourse exists in which climate change is seen as an outcome of the way humans live, with recognition of the link between environmental and human health, meaning hospitals and healthcare must consider both to be most effective in the long term (27, 28). A value-based model has been proposed to deliver healthcare through a planetary health lens (29). Value in healthcare has been described as “the measured improvement in a patient's health outcomes for the cost of achieving that improvement” (30). To date, healthcare services consider environmental sustainability independently of value-based healthcare (31). That is to say, the environmental cost that comes from the natural resources used and the waste and emissions created when treating a patient is not factored into this equation. Instead, healthcare's carbon footprint should be recognized as a cost in the value equation (31, 32). As such, the design and delivery of care is based on both patient outcomes and associated environmental impacts, in line with planetary health. To achieve this, internal systems and structures must be set up to support sustainability initiatives. This includes strategies demonstrated by the exemplar hospitals (Figure 1) such as embedding sustainability into the hospital mission and vision, setting up accountability mechanisms, long term strategic decision making, creating dedicated working groups and leadership that encourage staff to drive improvement. Another example is including sustainability as an indicator of care quality (33).

Finally, the regulative pillar highlighted the contrast between how staff from exemplar and business-as-usual hospitals see external influences such as regulation and member organizations. In relation to government policy, there was a sense of feeling let down because the “unicorn policy” had not emerged and constricted by the policy that did exist. In comparison, in the absence of desired policy, exemplar organizations advocated for what they wanted, or filled the gap with internal organizational policies. This signifies the need for a uniform and coherent public policy approach for sustainable foodservice with content considering evidence-based recommendations addressing all aspects of the food supply chain, including procurement, preparation, consumption and waste management. It is important that policy does not solely address food waste, as this is the low hanging fruit at the end of the food supply chain and encourages a narrow view of the issue as identified by the business-as-usual hospitals.

Sustainable foodservice policy needs to be part of a wider public policy approach to sustainable and net zero healthcare. To achieve this change, there is a need for a coordinated well-funded national level unit responsible for driving practice and policy change. As an example, the National Health Service in the UK has a sustainable development unit with many learnings that can be leveraged (34). This unit's work communicates the health risks of climate change, tackles issues to improve population health that also reduces environmental impact, and educates about sustainable practice in health service delivery (34).

Many exemplar hospitals were actively part of member organizations, both to seek guidance from and contribute to. More broadly, hospitals should take part in member organizations such as Global Green and Healthy Hospitals, Nourish Network and the Soil Association to aid their implementation of sustainable foodservice strategies. These organizations provide resources, guidance and networks to support implementation in the absence of national policy directive. For this reason, future policy initiatives and government approaches could leverage these organizations and work with them to promote their membership and resources to health services.

This research study has effectively compared two groups of hospitals at different stages of the sustainability phase model. It is important to note that there was less geographical diversity of hospitals in the business-as-usual group than the exemplar group and that interviews were conducted at different times, especially in relation to the coronavirus pandemic. Nonetheless, adopting the pillars of institutions to compare hospitals is a useful strategy to better understand the functioning of hospitals.

Hospitals can be stewards of change for advancing planetary and human health, both within their hospital and beyond. This study has identified several key differences in the pillars of institutions between hospitals in earlier and later stages of the sustainability phase model for achieving environmentally sustainable foodservices. Reconsidering and optimizing the pillars of institutions, including the knowledge and beliefs of staff, the culture and values of the hospital, and the policies, rules and regulations can build momentum for change.

## Data availability statement

The datasets presented in this article are not readily available because the authors did not receive consent for sharing data. Requests to access the datasets should be directed to SC, stefanie.carino@monash.edu.

## Ethics statement

The studies involving human participants were reviewed and approved by Monash University Human Research Ethics Committee. The patients/participants provided their written informed consent to participate in this study.

## Author contributions

SC undertook investigation and formal analysis and wrote the original draft. JP and JC verified coding and interpretation. All authors were involved in conceptualization and methodology and contributed to reviewing and editing.

## Funding

SC was supported by a Monash University Postgraduate Publications Award.

## Conflict of interest

The authors declare that the research was conducted in the absence of any commercial or financial relationships that could be construed as a potential conflict of interest.

## Publisher's note

All claims expressed in this article are solely those of the authors and do not necessarily represent those of their affiliated organizations, or those of the publisher, the editors and the reviewers. Any product that may be evaluated in this article, or claim that may be made by its manufacturer, is not guaranteed or endorsed by the publisher.
